# Biological Stability and Microbial Recovery Responses in Vermicomposting of Chemically Intensive Tomato Residues: Defining Management Limits

**DOI:** 10.3390/toxics14020129

**Published:** 2026-01-29

**Authors:** Fevziye Şüheda Hepşen Türkay

**Affiliations:** Department of Soil Science and Plant Nutrition, Agricultural Faculty, Kırşehir Ahi Evran University, 40100 Kırşehir, Türkiye; suheda.turkay@ahievran.edu.tr; Tel.: +90-386-2804816

**Keywords:** biological response, *Eisenia fetida*, pesticide stress recovery, vermicomposting, vermistabilization, tomato residues

## Abstract

The intensive cultivation of greenhouse tomatoes generates massive quantities of vegetative residues often laden with potentially complex pesticide contaminants, posing a dual challenge of waste management and environmental toxicity. This study investigated the biological feasibility and system tolerance of valorizing these hazardous residues through vermicomposting with *Eisenia fetida*, using mixtures of cattle manure and tomato residues (TR) at varying ratios (0–60%) over a 45-day incubation period. The process was monitored through physicochemical parameters (pH, EC, C/N ratio) and sensitive biological indicators (Basal Respiration and Microbial Biomass Carbon). While TR inclusion rates exceeding 30% induced acute inhibitory effects (100% mortality within 5 days) due to acute toxicity, mixtures containing up to 30% were successfully processed. The biological monitoring revealed a distinct “biphasic response”: an initial “metabolic lag phase” (days 0–15) driven by chemical stress, followed by a robust “biological recovery” where microbial activity surged significantly after day 30. Correlation analyses confirmed that this recovery was mechanically linked to the acidification of the substrate, as indicated by strong negative correlations between pH and biological activity (*r_s_* = −0.70). Ultimately, vermicomposting significantly reduced Electrical Conductivity (EC) and lowered the C/N ratio below 15 in all viable treatments, confirming the stabilization of waste into an agronomically mature product. The results demonstrate that the earthworm gut functions as an effective bioreactor, facilitating biological stabilization and the mitigation of toxicity in pesticide-laden biomass. This study concludes that vermicomposting is a robust strategy for converting toxic agro-wastes into a stabilized organic amendment, provided that the residue load is managed within the identified physiological tolerance threshold of 30%.

## 1. Introduction

The pressures of climate change and the progressive degradation of arable land due to intensive anthropogenic activities necessitate a shift towards more sustainable agricultural practices to ensure soil security for future generations. The indiscriminate use of chemical fertilizers and pesticides has severely depleted soil organic matter and diminished biological vitality [1,2,3], posing a significant threat to long-term food security [4]. Consequently, there is a global paradigm shift aimed at replacing synthetic inputs with organic amendments derived from the valorization of agricultural by-products [5]. In terms of agricultural sustainability, the recycling of organic material lost from the soil not only preserves soil fertility but also optimizes the nutrient balance that determines the industrial quality and phytochemical content of all crops, including medicinal and aromatic plants [6,7,8].

Among these by-products, residues from tomato cultivation (specifically vegetative biomass comprising leaves and stems, excluding roots and fruits) represent a massive waste stream. Tomato stands as the most widely cultivated vegetable crop in protected agriculture (greenhouses) worldwide, with global production exceeding 180 million tons annually [9,10,11]. This intensive production model inherently generates vast quantities of vegetative biomass residues. Currently, these humid post-harvest residues are often managed unsustainably through incineration or uncontrolled dumping. Direct soil applications of these raw residues are agronomically inefficient due to their high C/N ratio (approx. 51:1), which triggers nitrogen immobilization. Unlike traditional composting, vermicomposting is intended to produce a material with higher fertilizer value and a significantly lower C/N ratio. Generally, raw organic materials require at least a pre-composting phase to mitigate disadvantageous properties before soil incorporation. Direct application of such residues, especially those with high pesticide loads, threatens the sustainability of soil health due to prolonged decomposition times and toxicity risks. Therefore, determining the vermicomposting limits of these specific materials is the primary objective of this study.

The environmental fate of these potentially pesticide residues is a growing concern. It is critical to note that conventional composting, while effective for stabilizing organic matter, often fails to degrade recalcitrant pesticide molecules. This limitation has been recognized for decades [12], demonstrated in full-scale assessments [13], synthesized in comprehensive reviews [14,15], and recently provided with definitive evidence in high-resolution field studies [16]. Thus, the toxicity is not necessarily reduced during the process. Consequently, even if legally composted, chemically intensive greenhouse residues pose a risk of “secondary soil contamination.” When such contaminated biomass is incorporated into the soil, toxic compounds can inhibit the enzymatic activities of native soil microbiota, disrupt nutrient cycling, and decrease overall microbial diversity. Specifically, residues of fungicides and insecticides commonly used in greenhouse agriculture are known to persist in the environment, posing a chronic threat to non-target organisms.

Therefore, the safe recycling of these hazardous matrices requires a stabilization process that extends beyond simple decomposition. Vermicomposting, a bio-oxidative process driven by the synergistic interaction between earthworms (*Eisenia fetida*) and microorganisms, offers a superior biological advantage [17,18,19,20]. Dominguez and Edwards [21] characterize the earthworm gut as a natural ‘bioreactor,’ where ingested organic matter is mechanically fragmented, moistened, and inoculated with microbial-rich mucus. This intense biological activity distinguishes vermicomposting from conventional methods, enabling the bioaccumulation of heavy metals and accelerating the biodegradation of organic pollutants through enzymatic pathways [22,23].

Although numerous studies have successfully utilized *Eisenia fetida* to degrade various agro-industrial wastes—ranging from olive mill wastewater to sewage sludge—research specifically targeting phytotoxic and chemically treated vegetative residues remains limited [24,25,26]. Previous trials have often focused on pre-treated or low-toxicity waste. However, the direct vermicomposting of fresh, chemically treated greenhouse residues presents a unique challenge due to the potential for acute neurotoxic effects on earthworms. Nevertheless, *Eisenia fetida* is equipped with a robust enzymatic stress mitigation system—primarily involving glutathione S-transferases (GSTs) and cytochrome P450 monooxygenases—that facilitates the metabolic breakdown of xenobiotics at sub-lethal concentrations [27,28].

Rather than focusing solely on chemical degradation, the objective was to determine the functional resilience of the vermicomposting process when subjected to varying loads of potentially phytotoxic tomato residues. This study was designed to evaluate the feasibility of converting these toxic wastes into a safe soil amendment and to identify the optimum mixing ratio. It was hypothesized that while high residue loads might initially suppress metabolic activity, there exists a critical inclusion level and a transitional phase where the system shifts from chemical inhibition to active microbial proliferation. By monitoring these dynamics, this research aims to define the physiological tolerance thresholds for safe tomato residue management. To test this, the process was monitored using sensitive biological indicators, specifically Basal Respiration (BR) and Microbial Biomass Carbon (MBC), to assess the eco-physiological response of the system under chemical stress.

## 2. Materials and Methods

### 2.1. Raw Materials and Preparation

In this study, tomato plant residues (TR), cattle manure (CM), and the earthworm species *Eisenia fetida* were utilized as primary research materials. To simulate a realistic waste management scenario under high-chemical input conditions, vegetative residues were collected from tomato plants cultivated in a soilless geothermal greenhouse unit at Kırşehir Ahi Evran University. These plants had been subjected to intensive pesticide applications during the cultivation cycle. Specifically, the chemical pest management regime employed was representative of common greenhouse management protocols, including classes such as neonicotinoids (e.g., Imidacloprid, Deltamethrin) and synthetic pyrethroids and systemic fungicides to control common greenhouse pests and fungal pathogens.

Following the fruit harvest, the fresh vegetative biomass was collected, chopped into smaller pieces, and air-dried in the shade under cool, dry conditions to preserve its chemical integrity. To ensure standardization and homogeneity, the dried residues were ground using a laboratory mill and passed through a 1 mm sieve.

Cattle manure was selected as the basal bedding material and carrier substrate for the earthworms. This material was sourced from the Vermiculture Facility of Kırşehir Ahi Evran University. Prior to use, the manure underwent a pre-composting process to eliminate volatile gases and excess heat, after which it was air-dried and sieved through a 1 mm mesh. The epigeic earthworm species, *Eisenia fetida*, was also obtained from the same facility.

To prepare the vermicomposting feedstocks, the processed tomato residues and cattle manure were mixed thoroughly to ensure a homogeneous texture, facilitating uniform consumption by the earthworms. The experiment was established and conducted in 5 L plastic pots.

### 2.2. Experimental Strategy and Treatment Mixtures

The experimental strategy was designed to focus on the “process efficiency” and biological response of the vermicomposting system rather than direct toxin quantification. Based on the hypothesis that earthworm-mediated stabilization facilitates microbial resilience, biochemical parameters (e.g., C/N ratio, respiration) were selected as the primary indicators to determine the “optimum mixing ratio/management limits” where toxic waste does not inhibit the process.

Preliminary studies and literature data indicate that earthworms typically cannot survive in substrates where pesticide-heavy or phenolic-rich material inclusion exceeds 50–60%. Therefore, to identify the critical tolerance threshold, seven different mixing ratios were established. The experimental treatments were prepared by blending the processed tomato residues (TR) and cattle manure (CM) on a dry weight basis (*w*/*w*) to achieve the target mixing ratios. The experimental treatments are detailed in Table 1.

### 2.3. Vermicomposting Setup and Process Maintenance

The prepared mixtures were placed into 5 L plastic experimental pots. Each pot was filled with 3 kg of the substrate mixture (calculated on a dry weight basis). Fifty clitellated (adult) *Eisenia fetida* earthworms, with an average individual weight of approximately 1.5 g, were inoculated into each pot.

The duration of the experiment was set at 45 days, determined by the calculated daily feeding rate of the earthworm population and the substrate volume. Sampling was conducted periodically to monitor the temporal evolution of the process; sub-samples were collected from all treatments on days 0, 7, 15, 30, and 45.

Throughout the incubation period, the pots were weighed daily to monitor moisture loss. The moisture content was maintained at a constant level (approximately 70–80% of the water-holding capacity) by adding distilled water as needed. To ensure accuracy, the maximum water-holding capacity for each mixture was pre-determined using the saturation paste method prior to the start of the experiment.

### 2.4. Experimental Design and Sampling Methodology

The experiment was arranged in a Completely Randomized Design (CRD) with three replications. A total of 84 independent experimental pots were established (7 treatment ratios × 4 sampling intervals × 3 replicates) to allow for destructive sampling. At each designated sampling interval (Days 7, 15, 30, and 45), a specific set of 21 pots (one per treatment per replicate) was terminated and processed for analysis. This approach ensured that the vermicomposting process in each pot remained undisturbed by prior sampling activities, maintaining the physical integrity and moisture stability of the substrate throughout the incubation. Initial characteristics (Day 0) were determined from the prepared mixtures prior to their placement in the pots and inoculation with earthworms.

The incubation was conducted in a controlled plant growth chamber under stable environmental conditions, with a fixed temperature of 22 ± 1 °C and controlled photoperiod to prevent light-induced stress on the earthworms. Specific sampling protocols were strictly followed to ensure data integrity.

Fresh sub-samples for biological analyses were immediately sieved (<2 mm) and stored in sealed polyethylene bags at 4 °C to preserve microbial viability and enzymatic activity until processing (within 24 h). Samples for chemical analyses were air-dried at room temperature, ground, and passed through a 2 mm sieve for general physicochemical characterization (pH, EC) and a 0.5 mm sieve for total nitrogen and organic matter analysis.

### 2.5. Chemical and Biological Analyses

The chemical and biological properties of both the initial raw materials (tomato residues and cattle manure) and the final vermicompost products were determined using standard analytical procedures. Additionally, biological activity was monitored via respiration-based methods. The specific methodologies and references are detailed in Table 2.

Microbial biomass carbon (MBC) was estimated to use the substrate-induced respiration (SIR) method as described by Anderson and Domsch [32]. Briefly, moist samples (equivalent to 10 g dry weight) were amended with a powder mixture containing 40 mg of glucose to trigger maximum respiratory response. The CO_2_ production rate was monitored hourly over a 4 h incubation period using the titrimetric approach [31]. MBC was then calculated based on the maximum initial respiratory response using the conversion equation (MBC = 40.04 × mg CO_2_ g^−1^ + 3.75) and results were expressed as mg C g^−1^ dry substrate.

Basal respiration (BR) was measured to determine the metabolic activity under field capacity without exogenous carbon addition. Fresh samples were incubated at 22 °C for 24 h in hermetically sealed systems. The evolved CO_2_ was trapped in an alkaline solution (Ba (OH)_2_·8H_2_O and BaCl_2_), and the residual hydroxide was quantified by titration with standardized hydrochloric acid (HCl) using phenolphthalein as a pH indicator. The BR data were reported as mg CO_2_ g^−1^ dry substrate.

### 2.6. Statistical Analyses

The experiment was arranged in a factorial completely randomized design with 7 treatment mixtures (including a 0% tomato residue control) and 4 incubation times, with 3 replicates each (7 treatments × 4 sampling intervals × 3 replicates = 84 units). Due to severe earthworm mortality at the highest tomato residue doses (50% and 60% TR by day 1, and 40% TR by day 5), statistical comparisons focused on the four surviving treatments (0%, 10%, 20%, 30% TR). All data were analyzed using GraphPad Prism 8 software. A two-way analysis of variance (ANOVA) was performed to assess the effects of treatment (tomato waste application rate) and time (incubation day) on each measured parameter, as well as their interaction.

Where the interaction term was significant, post hoc analysis was performed using Tukey’s multiple comparisons test (Tukey’s HSD post hoc test for analyzing simple effects) to compare treatment means within each specific time point, utilizing the pooled error variance from the main Two-Way ANOVA. In one case, to compare final-day values among treatments (e.g., pH on day 45), a separate one-way ANOVA was applied for that time point alone, with Tukey’s post hoc test, as justified by the analysis objectives. All statistical differences were evaluated at a significance level of *p* < 0.05. Additionally, a Spearman rank correlation matrix (non-parametric) was computed across all treatments and time points (*n* = 48) to evaluate relationships among chemical and biological parameters. Results are reported as significant when *p* falls below the 0.05 threshold, and exact *p* values or adjusted *p* values are provided for key comparisons to indicate the strength of differences.

## 3. Results

### 3.1. Earthworm Survival and Experimental Adjustment

As detailed in Section 2, the experiment initially included seven mixtures with varying tomato residue (TR) concentrations. However, a critical physiological threshold was observed regarding earthworm survival. In treatments containing 40%, 50%, and 60% TR, the earthworms (*Eisenia fetida*) failed to survive beyond the first few days of exposure. A sharp distinction in survival was observed based on the mixing ratio. While treatments with <30 TR showed 0% mortality throughout the 45-day period, higher doses exceeded the tolerance threshold. Specifically, the 50–60% TR groups exhibited immediate mortality within the first 24 h, whereas the 40% TR group survived for 5 days before total population collapse, indicating a rapid onset of acute toxicity beyond the 30% limit. The experiment proceeded and data were reported only for the viable mixtures: the Control (0% TR), 10% TR, 20% TR, and 30% TR.

### 3.2. Chemical Properties

The chemical properties of the cattle manure and tomato residues used in the experiment are presented in Table 3. Throughout the 45-day vermicomposting period, the effects of incubation time and tomato residue proportion on chemical parameters were evaluated, revealing significant variations across all treatments.

The chemical profiles of cattle manure (CM) and tomato residues (TR) exhibited contrasting chemical properties, establishing the baseline for the vermicomposting process. CM functioned as a balanced basal medium characterized by a near-neutral pH and moderate salinity levels. In contrast, TR was identified as a carbon-dense substrate with a more acidic profile and higher electrical conductivity. Throughout the 45-day vermicomposting period, the chemical composition of the viable mixtures (0–30% TR) underwent significant transformation. Two-way ANOVA results confirmed that these changes were not merely time-dependent but were also significantly influenced by the interaction between the TR application rate and incubation duration. The process followed a consistent trajectory toward acidification, reduced salinity, and increased nitrogen concentration, reflecting the biological cooperation of *Eisenia fetida* with the microbial community.

#### 3.2.1. pH Dynamics

The pH values exhibited a statistically significant decline over time in all treatments, with a notable interaction between treatment and time (*p* = 0.0247). Initially (Day 7), pH values were slightly alkaline and statistically similar across all groups, ranging around 7.6. However, as the process progressed, the biodegradation of organic matter led to acidification. By the end of the incubation (Day 45), the pH dropped to a range of approximately 7.20–7.35.

**Figure 1:**
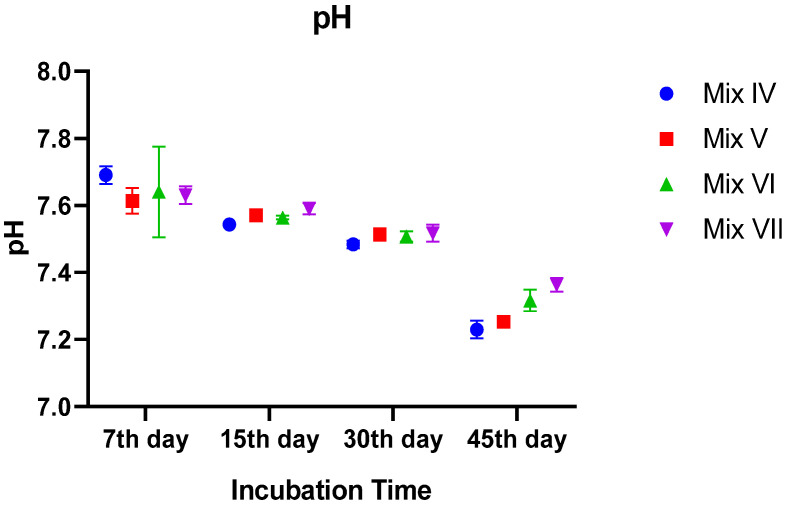
Temporal changes in pH values of vermicomposts during the 45-day incubation period. Vertical bars represent standard error (SE).

A clear dose-dependent effect was evident in the final stage. The mixture with the highest tomato residue (30% TR) exhibited the lowest final pH (~7.20), which was significantly lower than that of the Control and 10% TR groups (~7.35, *p* < 0.01). The mixture with 30% TR resulted in the most pronounced reduction in pH compared to pure manure (Figure 1).

#### 3.2.2. Electrical Conductivity (EC)

Electrical conductivity (EC), representing the soluble salt content, showed a marked decrease in every treatment over the 45-day period. Two-way ANOVA revealed a highly significant interaction between treatment and incubation time (*p* < 0.0001), meaning the pattern of salt reduction varied among the mixtures.

**Figure 2:**
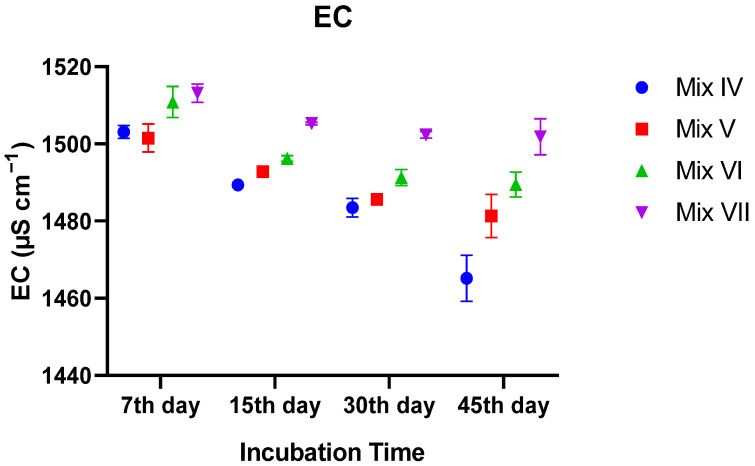
Temporal changes in electrical conductivity (EC) of vermicomposts under different tomato residue (TR) application rates. Values are mean ± SE.

Although all mixtures started with similar EC levels (~1500 µS cm^−1^), significant differences emerged rapidly. By Day 45, the control group (0% TR) retained the highest EC value (~1502 µS cm^−1^), whereas the 30% TR treatment dropped to the lowest level (~1465 µS cm^−1^). The pairwise comparisons at the end of the study confirmed that each treatment was statistically distinct (*p* < 0.05), demonstrating that incorporating tomato residue significantly reduced the final salinity of the vermicompost relative to the control (Figure 2).

#### 3.2.3. Organic Matter (OM) and Organic Carbon (OC)

The initial organic matter content was naturally higher in the treatments amended with tomato residue, reflecting the high organic load of the plant waste. Over the course of vermicomposting, OM declined significantly in all groups due to mineralization (*p* ~ 0.046).

**Figure 3:**
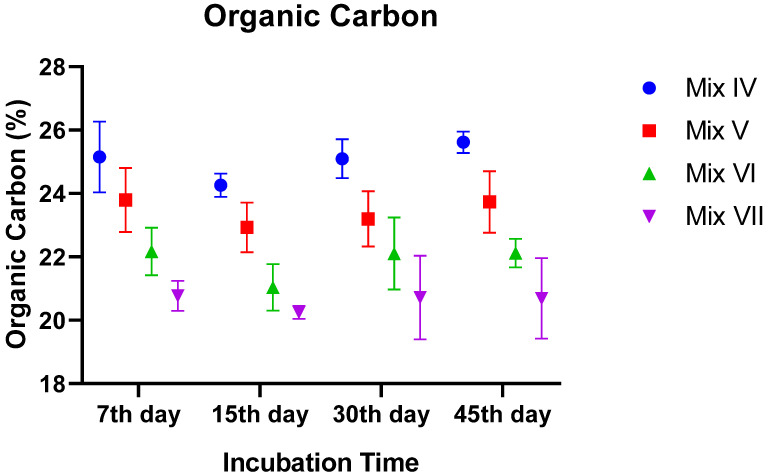
Variation in organic carbon (OC) content of vermicomposts during incubation as influenced by tomato residue (TR) content.

Despite the general loss of mass, the treatment effect remained highly significant (*p* < 0.0001) throughout the process. The 30% TR mixture consistently maintained the highest OM percentage. By Day 45, the vermicompost derived from 30% TR still contained approximately 44% OM, whereas the Control (0% TR) had decreased to ~36%. Organic Carbon (OC) followed an identical trend, showing a gradual decline with time but remaining significantly higher in TR-amended groups compared to the control (*p* < 0.0001) (Figure 3).

#### 3.2.4. Total Nitrogen (TN)

Total Kjeldahl nitrogen exhibited a progressive increase over time in all experimental sets (*p* = 0.0001). This enrichment is consistent with the concentration effect resulting from the loss of carbon as CO_2_.

**Figure 4:**
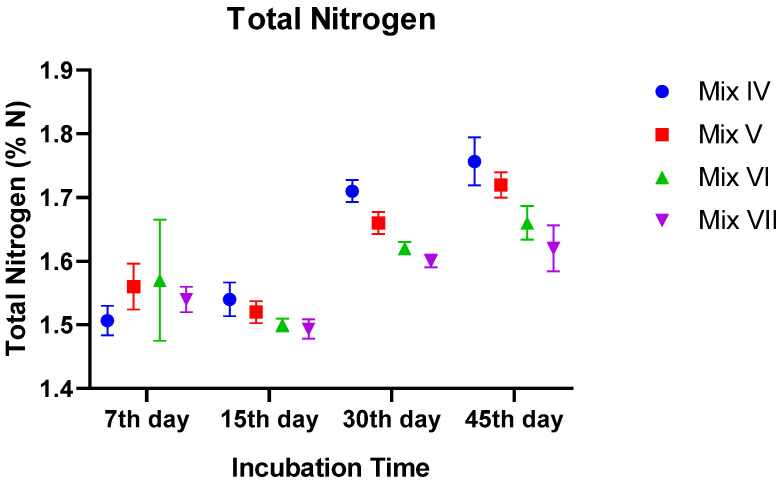
Temporal changes in total nitrogen (TN) content of vermicomposts. Values are mean ± SE.

A significant interaction (*p* < 0.01) was observed between the residue rate and time. In the early stages (Days 7 and 15), TN percentages were statistically equivalent across groups (~1.50–1.57%). However, distinct differences manifested by Day 30 and solidified by Day 45. The 30% TR treatment accumulated the highest nitrogen content, reaching ~1.76% N, which was significantly greater than the control group (~1.62% N; *p* < 0.001). The 20% TR group also finished with significantly higher nitrogen (~1.72%) compared to the control. These results suggest that tomato residue incorporation enhances the nitrogen value of the final product (Figure 4).

#### 3.2.5. C/N Ratio

The C/N ratio, a key indicator of compost maturity, decreased sharply across all treatments (*p* < 0.0001). The control group started with a C/N ratio of ~13.5 and finished at ~12.8, while the 30% TR mix decreased from ~16.7 to ~14.6.

**Figure 5:**
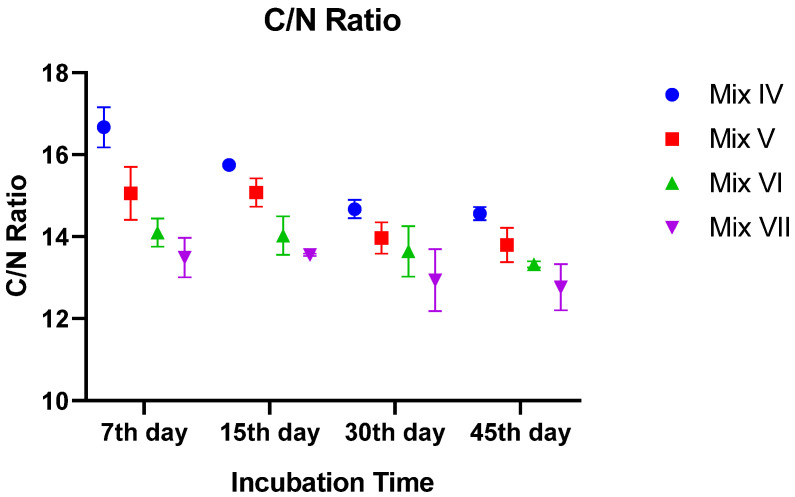
Temporal variation in C/N ratio of vermicomposts across the incubation periods. Values are mean ± SE.

Although the addition of tomato residue resulted in higher C/N ratios throughout the process due to the initial carbon load, all treatments successfully reached maturity values. By the end of incubation, even the highest residue mixture (30% TR) achieved a C/N ratio below 15, confirming the stabilization of the waste (Figure 5).

### 3.3. Biological Activity

The biological response of the system was evaluated through Basal Respiration (BR) and Microbial Biomass Carbon (MBC). Both parameters showed low initial values followed by a dramatic increase, reflecting the proliferation of the decomposer community. However, the presence of pesticide-laden tomato residue exerted a clear suppressive effect on this biological resurgence.

#### 3.3.1. Basal Respiration (BR)

**Figure 6:**
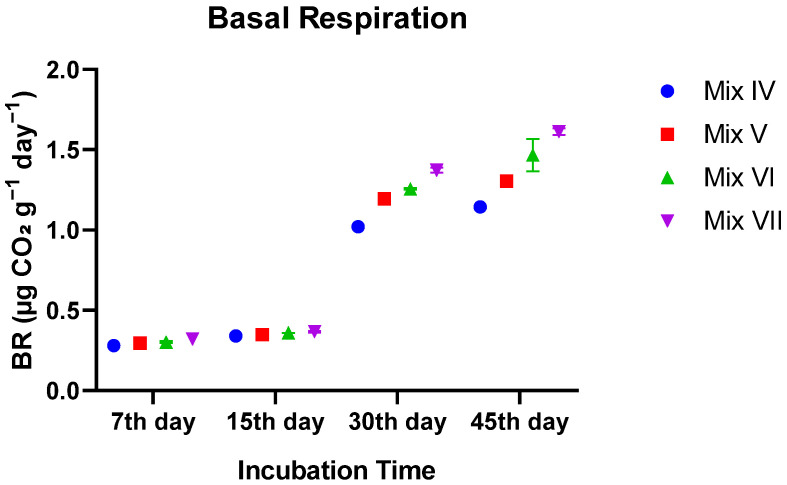
Temporal evolution of basal respiration (BR) in vermicomposts as influenced by tomato residue (TR) content. Values are mean ± SE.

Basal respiration increased significantly with time (*p* < 0.0001), but the rate of increase was strongly dependent on the treatment. During the first 15 days, respiration rates were low and similar among groups. From Day 30 onward, a significant divergence occurred. The Control group (0% TR) exhibited the highest metabolic activity, reaching ~1.61 µg CO_2_ g^−1^ day^−1^ by Day 45. In contrast, the 30% TR mixture showed significantly inhibited respiration, reaching only ~1.14 µg CO_2_ g^−1^ day^−1^ (*p* < 0.0001). The 10% and 20% TR groups displayed intermediate values, confirming a dose-dependent toxic effect on microbial metabolism (Figure 6).

#### 3.3.2. Microbial Biomass Carbon (MBC)

**Figure 7:**
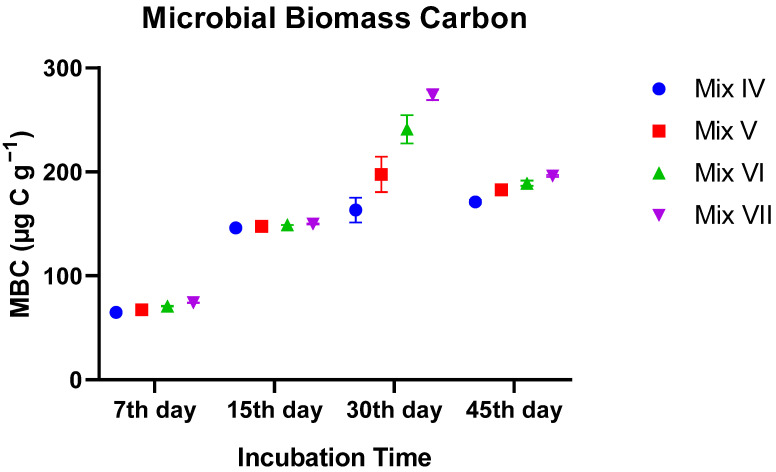
Changes in microbial biomass carbon (MBC) of vermicomposts during incubation. Values are mean ± SE.

The MBC data mirrored the respiration trends. A highly significant treatment-by-time interaction (*p* < 0.0001) was detected. While all groups showed an increase in biomass over time, the Control group exhibited the most pronounced peak in biomass. By Day 30 and 45, the Control group achieved the highest microbial biomass (~196 µg g^−1^), whereas the 30% TR treatment remained significantly lower (~171 µg g^−1^). This indicates that higher tomato residue content curtailed microbial biomass accumulation, likely due to the toxic compounds present in the residue (Figure 7).

### 3.4. Interrelationships Between Physicochemical and Biological Parameters

**Figure 8:**
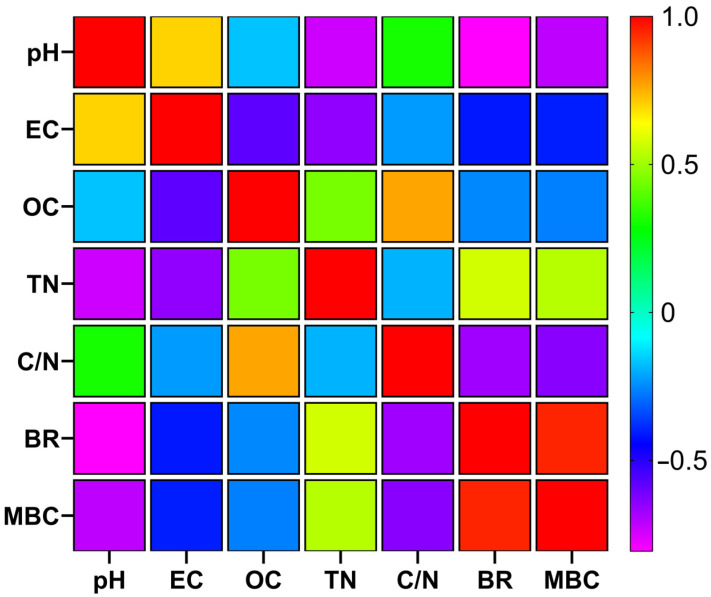
Spearman correlation matrix heatmap showing the relationships between chemical and biological parameters (N = 48). Positive correlations are indicated in warm colors (reds), and negative correlations in cool colors (blues).

A Spearman correlation analysis was conducted to elucidate the relationships between the chemical and biological parameters investigated (Figure 8). The results revealed a nearly perfect positive correlation between BR and MBC (*r_s_* = 0.948, *p* < 0.0001), confirming that biomass growth and metabolic activity occurred in tandem throughout the process. In terms of chemical influences, strong negative correlations were observed between pH and biological markers, specifically for BR (*r_s_* = −0.807) and MBC (*r_s_* = −0.714), suggesting that microbial activity intensified as the pH moved toward more neutral or acidic levels. Furthermore, the system’s maturity was reflected in the C/N ratio, which showed an inverse relationship with BR and MBC, while maintaining a strong positive correlation with OM (*r_s_* = 0.764, *p* < 0.0001). These associations directly link the reduction in organic matter and the decline of the C/N ratio to the sustained biological activity within the system.

While the correlation matrix provided a comprehensive overview of the associations, linear regression analyses were further conducted on the most influential predictor-response pairs to elucidate the mechanistic drivers of the vermicomposting process.

**Figure 9:**
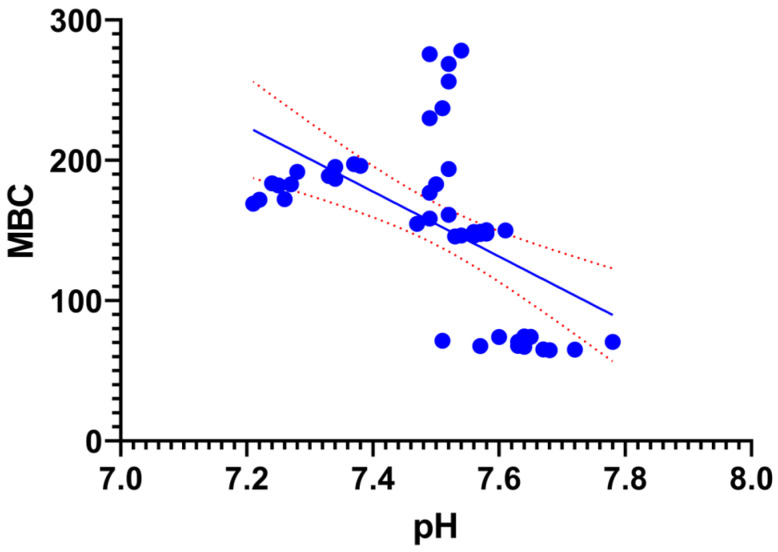
Linear regression analysis between substrate pH (X-axis) and microbial biomass carbon (MBC) (Y-axis) throughout the vermicomposting period. The solid line represents the calculated regression trend (*Y* = −231.5*X* + 1890), and the dashed lines indicate the 95% confidence intervals (*R*^2^ = 0.294, *p* < 0.0001).

The regression analysis revealed an extremely significant negative linear relationship between pH and MBC (*p* < 0.0001) (Figure 9). This negative slope (Slope = −231.5) statistically confirms that the gradual acidification of the substrate, moving from alkaline towards more neutral levels, acted as a primary driver for microbial recovery. The model indicates that approximately 29.4% of the variation in microbial biomass can be explained solely by the shift in pH, highlighting the critical role of chemical stabilization in mitigating initial toxicity.

**Figure 10:**
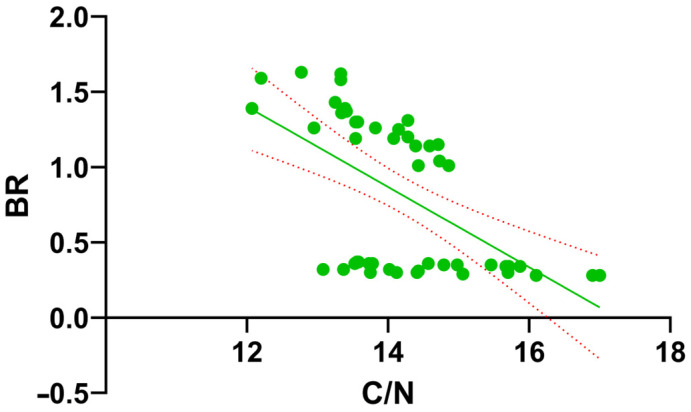
Linear regression analysis between the C/N ratio (X-axis) and basal respiration (BR) (Y-axis). The solid line represents the calculated trend (*Y* = −0.2667*X* + 4.602), and the dashed lines indicate the 95% confidence intervals (*R*^2^ = 0.323, *p* < 0.0001).

Furthermore, the relationship between substrate maturation and metabolic activity was evaluated through a second linear regression analysis between the C/N ratio and basal respiration (BR) (Figure 10). The results demonstrated a highly significant negative linear association (*p* < 0.0001, *R*^2^ = 0.323), confirming that the narrowing of the C/N ratio was a fundamental driver for the observed biological recovery phase. The regression model (*Y* = −0.2667*X* + 4.602) statistically validates that as the organic matter reached a more stabilized form (lower C/N ratio), the system’s metabolic rate significantly intensified. This trend provides robust evidence that biological stability and microbial resurgence in pesticide-laden residues are synchronized processes strictly linked to the chemical evolution of the composting matrix.

**Figure 11:**
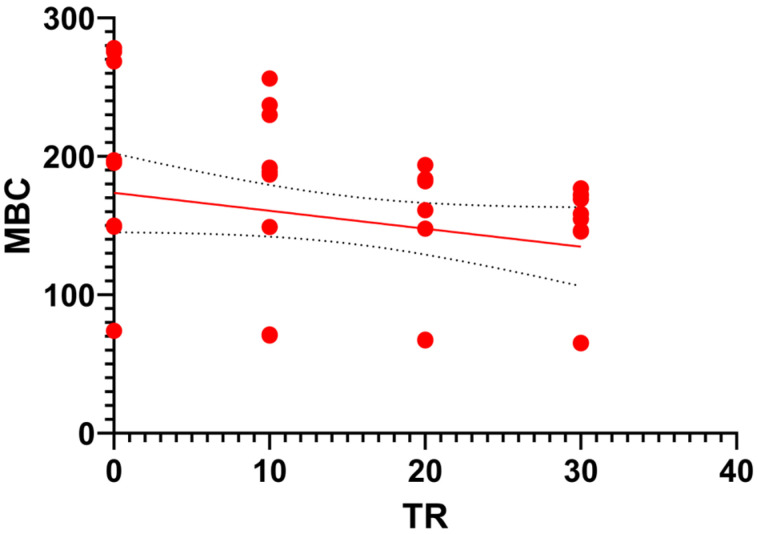
Linear regression analysis between the tomato residue (TR) ratio (X-axis) and microbial biomass carbon (MBC) (Y-axis) pooled across all incubation intervals (*N* = 48). The solid line represents the general trend (*Y* = −1.298*X* + 173.7), showing a marginal but non-significant dose–response effect (*R*^2^ = 0.06, *p* = 0.0934).

Lastly, a linear regression analysis was performed to evaluate the overall dose–response relationship between the tomato residue (TR) inclusion ratio (0–30%) and microbial biomass carbon (MBC) across the entire experimental period (Figure 11). The regression followed the equation: *Y* = −1.298*X* + 173.7, with a coefficient of determination (*R*^2^ = 0.06) and a *p*-value of 0.0934. While the negative slope indicates a marginal downward trend in microbial capacity as TR levels increase, the lack of statistical significance (*p* > 0.05) when pooling all time points suggests that the system’s temporal recovery phase eventually overshadows the initial dose-dependent inhibition. This indicates that within the viable range of 0–30%, the vermicomposting process maintains a relatively consistent biological functionality regardless of the residue load.

## 4. Discussion

Since vermicomposting systems are confined environments, the chemical composition of the waste pile is the most critical factor directly determining earthworm activity and process efficiency [33]. This study investigated the management of pesticide-laden greenhouse tomato residues (TR). The most significant toxicological finding was that TR inclusion rates exceeding 30% caused acute toxicity, rendering earthworm survival impossible. This indicates that the pesticide load surpassed the physiological tolerance limits of the earthworms [34]. However, in mixtures of 30% and below, cattle manure acted as a buffer, minimizing the toxic concentration and ensuring earthworm survival [33]. This buffering capacity suggests that the organic matrix of cattle manure effectively reduces the bioavailability of potentially pesticide residues, preventing the immediate collapse of the vermicomposting system at moderate TR inclusion rates.

The acute and high earthworm mortality observed in mixtures containing >30% tomato residues (TR) suggests that the limiting factor is chemical toxicity inherent to the waste load, rather than natural ‘inedibility’ or nutrient deficiency. Admittedly, while specific pesticide residue analysis would have provided direct confirmation, the primary focus of this study was to determine the biological manageability limits of the aggregate waste stream rather than tracking specific active ingredients. However, the drastic shifts in biological properties and the specific physiological stress responses of earthworms can only be plausibly explained by the presence of a toxic load when interpreted in light of existing literature.

This distinction is supported by comparative data. For instance, Pramanik, Ghosh [35] reported successful vermicomposting using 50% tomato vegetative waste mixed with cattle manure. However, their waste was derived from open-field conditions where environmental factors likely reduced residue loads, standing in stark contrast to the controlled greenhouse conditions with intensive agrochemical pressure used in our study. Consequently, the mortality threshold observed at >30% TR in our findings reflects a limitation driven by the agrochemical accumulation specific to the greenhouse production model, not the plant tissue itself. However, the changes in the biological properties of the medium and the physiological responses of the earthworms identified in this study are strongly supported by the literature and are specifically associated with the presence of pesticide load.

The non-significant regression (*p* = 0.0934) between TR ratio and MBC across the 45-day period further reinforces the ‘functional resilience’ of the system. While potential dose-dependent toxicity might be anticipated at varying residue loads, the statistical model suggests that once the system passes the initial ‘metabolic lag phase’ (days 0–15), the biological recovery is robust enough to reach a comparable microbial ceiling across all viable mixtures (0–30% TR). This implies that the 30% TR threshold is not just a limit of survival, but a point where the vermicomposting system effectively manages to normalize its biological activity despite the initial pesticide burden, eventually yielding a product with stable microbial biomass levels similar to the control group.

The significant decline in pH throughout the process is linked to intensified earthworm activity, as earthworm casts are near-neutral and actively regulate the substrate [36]. Additionally, organic acids released during organic matter mineralization contributed to the pH reduction [26]. Regarding salinity (EC), high TR treatments (30%) resulted in lower final EC values compared to the control. This inverse trend is attributed to the biodegradable organic matter in the waste stimulating microbial activity; the increased biomass likely assimilated soluble ions, thereby reducing salinity [37,38]. Since the closed system prevented physical leaching, this reduction was driven purely by biological mechanisms.

OM and OC contents decreased in all groups due to mineralization driven by the combined metabolic activity of earthworms and microorganisms [39,40]. However, high-TR treatments maintained significantly higher carbon levels compared to the control (*p* < 0.0001). This can be explained by earthworm fragmentation increasing the surface area, thereby facilitating microbial colonization [41]. Crucially, the high carbon retention suggests the formation of ‘microbial necromass’ [42]. As emphasized by Liang, Schimel [43], increased microbial anabolism, despite the presence of pesticides, transformed labile carbon into persistent forms, effectively stabilizing it within the system.

A distinct increase in TN content was observed across all treatments throughout the process. This enrichment is primarily attributed to the ‘concentration effect’ resulting from mass loss via carbon mineralization [44,45] and nitrogenous contributions (mucus, urine) from earthworm metabolic activity [46]. Notably, the 30% tomato residue mixture achieved the highest nitrogen content despite the pesticide load, proving that the nutritional value of the waste was conserved. Concurrently, the C/N ratio declined significantly in all groups. Although mineralization kinetics in high-residue treatments were relatively slower than the control due to potentially pesticide pressure, the final C/N ratio in all viable treatments successfully dropped below 15, the accepted threshold for mature compost [47,48]. This confirms that the system effectively stabilized the waste into an agronomically safe form despite toxic stress.

BR and MBC serve as the most sensitive indicators of the system’s eco-physiological status [49,50]. The low activity observed during the first 15 days points to acute inhibition of microbial metabolism by pesticide residues [51]. However, the sharp surge in both parameters from day 30 onwards demonstrated that the system entered a ‘recovery phase.’ This biological resurgence is attributed to the earthworm gut functioning as a ‘bioreactor’ to degrade contaminants and stimulating microbial activity through mucus secretion (priming effect) [52]. This biological resurgence suggests a potential degradation of the initial toxic compounds into less inhibitory intermediates, facilitated by the synergistic action of earthworms and the stimulated microbial community. The transition from the initial lag phase to a robust recovery phase confirms that the earthworm gut acts as an enzymatic bioreactor, not only stimulating microbial growth but potentially facilitating the transformation of toxic molecules into less inhibitory forms.

In the final stage (days 30–45), a distinct metabolic shift was observed: while microbial biomass declined, respiration continued to rise. This inverse relationship indicates a change in microbial strategy; under conditions of depleting nutrients and residual toxic stress, microorganisms diverted energy from ‘growth’ (anabolism) towards ‘maintenance and survival’ (catabolism) [53]. This metabolic shift highlights the ‘long-term energy cost’ of microbial resilience; the community prioritizes survival and detoxification pathways over biomass accumulation when faced with persistent chemical stressors in the tomato residues. Consequently, although high doses of tomato residue (30%) partially suppressed microbial growth, earthworm activity ensured the system was not sterilized but rather fostered a resilient and functional microbial community.

The Spearman correlation analysis empirically summarizes the interconnected chemical and biological dynamics governing the stabilization process. Specifically, the strong negative correlation between pH and biological activity parameters (BR and MBC) (*p* < 0.0001) proves that substrate acidification was not merely a chemical change but the primary driver enabling microbial revival. The significant negative correlation and the resulting regression model between pH and MBC provide robust evidence for the ‘acidification-driven biological recovery’ hypothesis in pesticide-stressed residues. In such complex vermicomposting systems, pH serves as a ‘master variable’ that governs both the microbial community structure and the chemical state of potential contaminants. The increase in MBC as pH declined suggests that the transition toward neutrality fulfilled a dual role: first, by providing a more optimal physiological environment for microbial proliferation, and second, by potentially influencing the degradation rate of residual pesticides through altered bioavailability. These findings demonstrate that the observed biological stabilization is a process strictly governed by the chemical evolution of the matrix, rather than a stochastic recovery. The strong negative correlation (*r_s_* = −0.70) indicates that vermicomposting facilitates a self-regulating stabilization process. Acidification serves as a biological trigger that optimizes the environment for pesticide-degrading microorganisms, thereby initiating the observed systemic recovery. Furthermore, the negative relationship between the C/N ratio and biological parameters confirms that organic matter stabilization proceeded simultaneously with biological recovery. The significant negative slope obtained from the C/N vs. BR regression (Slope = −0.2667) highlights the critical role of substrate maturation in overcoming initial chemical inhibition. In vermicomposting systems, a declining C/N ratio is the standard indicator of stabilization. Our findings show that this chemical stabilization acted as a ‘biological trigger’ for the recovery phase; as the more labile carbon fractions were processed and nitrogen was concentrated, the microbial community shifted from a state of acute inhibition toward robust metabolic activity. This causal interaction confirms that the earthworm-mediated narrowing of the C/N ratio provided the necessary physiological window for microbial recovery despite the presence of initial pesticide stress.

This study successfully demonstrated the biological feasibility of safely processing pesticide-laden tomato residues (TR) via vermicomposting, utilizing *Eisenia fetida*, provided the TR inclusion rate remains below the established acute toxicity threshold of 30%. The resultant vermicompost achieved agricultural maturity, confirmed by a final C/N ratio below 15 in all viable treatments. However, due to the scope of this investigation and current analytical constraints, the degradation kinetics and final concentrations of specific pesticide residues—which form the core of the ‘pesticide management’ opinion—could not be quantitatively presented for both the initial waste materials and the final processed product.

This limitation implies that the current findings primarily establish the biological viability of the system, setting critical, life-limiting operational thresholds (e.g., <30% TR). These established biological limits provide a vital foundation for future work.

Therefore, subsequent research efforts must focus on definitively assessing the removal efficiencies of the vermicomposting system on major pesticide classes (e.g., neonicotinoids, synthetic pyrethroids) using advanced analytical techniques (such as LC-MS/MS or GC-MS). Such comprehensive chemical analyses are necessary to conclusively confirm the full environmental safety profile of the final product for practical agricultural applications and to fully address the regulatory concerns related to residue management.

The shift towards neutrality fulfilled three critical functions: (I) Providing optimal conditions for microbial growth [54,55], (II) Enhancing pesticide biodegradability by altering their ionization state or reducing bioavailability [56], and (III) Stabilizing the nitrogen cycle by preventing ammonia toxicity [18]. Ultimately, biochemical stabilization (pH reduction and C/N narrowing) served as the essential prerequisite mechanism triggering biological recovery in this potentially pesticide-stressed environment.

## 5. Conclusions

This study successfully demonstrated the feasibility of valorizing potentially pesticide-contaminated greenhouse tomato residues through vermicomposting with *Eisenia fetida*. The findings provide critical insights into the operational limits and biological mechanisms required to transform this hazardous agro-waste into a safe and valuable soil amendment. The primary conclusion drawn from the toxicological response is that the survival of earthworms is strictly dose-dependent. A critical threshold was identified at a 30% inclusion rate; mixtures exceeding this level resulted in acute mortality, whereas ratios of 30% or lower supported a viable vermicomposting process. While direct quantification was not performed, the significant increase in microbial biomass and respiration during the second half of the process suggests a potential degradation of the chemical stressors, identifying 30% as the safe operational threshold for biological recovery. This defines the practical operational limit for utilizing fresh tomato waste in vermiculture systems without pre-treatment.

Biologically, the process exhibited remarkable resilience. Although the pesticide load initially induced an “acute metabolic inhibition,” evidenced by suppressed basal respiration and microbial biomass, the system successfully transitioned into a “recovery phase” after the second week. This restoration confirms the role of the earthworm gut as a “bioreactor” that facilitates the biological stabilization of the substrate and stimulates the re-colonization of the microbial community. The observed “biphasic response,” further supported by correlation analyses linking the recovery to substrate acidification (*r_s_* −0.70), provides a key mechanistic understanding of how earthworms manage chemical stress and initiate biological recovery in contaminated systems. From an agronomic perspective, the final product achieved high-quality standards within a relatively short period of 45 days. Despite the chemical stress, the process effectively stabilized organic matter, enriched the total nitrogen content through the “concentration effect,” and reduced salinity (EC) via biological assimilation. Most significantly, the C/N ratio dropped below 15 in all successful treatments, verifying that the final vermicompost reached a level of maturity safe for soil application.

In summary, this research confirms that vermicomposting is a robust strategy for the management of pesticide-laden tomato residues. It offers a sustainable, circular economy solution that not only mitigates the environmental risks associated with toxic agro-wastes but also converts them into a nutrient-rich bio-fertilizer, provided that the waste application rate is managed within the identified physiological tolerance limits of the earthworms. Ultimately, the final product transcends the definition of a mere fertilizer; it acts as a detoxified organic amendment capable of restoring microbial functional diversity and enhancing the resilience of agricultural soils against future chemical stressors.

For safe application approaches, commercial-scale vermicomposting facilities are advised to strictly adhere to the identified 30% threshold to ensure earthworm welfare and product quality. However, given that traditional composting facilitates the maturation and stabilization of organic matter, the integration of a pre-composting stage for tomato residues presents a promising avenue to potentially exceed this threshold. Future studies should focus on optimizing this pre-treatment to enhance waste recycling efficiency, while also elucidating the degradation kinetics of specific pesticide contaminants and identifying the microbial communities responsible for the significant biological recovery observed in the system.

## Figures and Tables

**Table 1:** Experimental treatments and mixing ratios (*w*/*w*, dry weight basis).

Groups	Substrate Composition (*w*/*w*)
Mix I	60% Tomato Residue + 40% Cattle Manure (60% TR + 40% CM)
Mix II	50% Tomato Residue + 50% Cattle Manure (50% TR + 50% CM)
Mix III	40% Tomato Residue + 60% Cattle Manure (40% TR + 60% CM)
Mix IV	30% Tomato Residue + 70% Cattle Manure (30% TR + 70% CM)
Mix V	20% Tomato Residue + 80% Cattle Manure (20% TR + 80% CM)
Mix VI	10% Tomato Residue + 90% Cattle Manure (10% TR + 90% CM)
Mix VII	0% Tomato Residue + 100% Cattle Manure (0% TR + 100% CM)

**Table 2:** The methods conducted for determination of chemical and biological properties of both the initial raw materials and the final vermicompost products.

Analysis	Methodology	Reference
Organic Matter (OM)	Loss on ignition (550 °C) after H_2_SO_4_ pretreatment	Ryan, Estefan [29]
Total Nitrogen (TN)	Modified Kjeldahl method (Velp Scientifica)	Bremner [30]
C/N Ratio	Calculated as (OM/1.724)/TN	Ryan, Estefan [29]
pH	Measured in a 1:10 (*w*/*v*) solid:distilled water suspension	Ryan, Estefan [29]
EC (Electrical Conductivity)	Measured in a 1:10 (*w*/*v*) solid:distilled water suspension	Ryan, Estefan [29]
Basal Respiration	CO_2_ evolution (Titrimetric measurement)	Anderson [31]
Microbial Biomass C	Substrate-induced respiration (SIR)—(glucose-induced respiration rate)	Anderson and Domsch [32]
Total Phosphorus (P)	Spectrophotometrically (yellow color method)	Ryan, Estefan [29]
Total Potassium (K)	Flame photometer	Ryan, Estefan [29]
Total Ca and trace elements (Fe, Cu, Zn, Mn)	Atomic Absorption Spectrophotometer (AAS)	Ryan, Estefan [29]

**Table 3:** Chemical analysis results of the cattle manure and tomato residue used in the experiment.

Analysis	Cattle Manure	Tomato Residue
pH 1:1	7.65	6.25
EC 1:1 (µS cm^−1^)	1523.56	2381.36
Organic Matter, %	35.866	88.743
Organic Carbon (C), %	20.734	51.470
Total Nitrogen, %	1.562	1.35
Available Phosphorus, %	2.533	0.96
Potassium, me 100 g^−1^	3.747	0.42
Ca + Mg, me 100 g^−1^	41.6	2.58
Calcium, %	1.895	1.92
Iron, ppm	4.62	447
Copper, ppm	3.72	381
Zinc, ppm	1.5361	153.25
Manganese, ppm	1.583	344

## Data Availability

The data presented in this study is available on request from the corresponding author.
